# Comparative Analysis of the Diversity of the Microbial Communities between Non-Fertilized and Fertilized Eggs of Brown Planthopper, *Nilaparvata lugens* Stål

**DOI:** 10.3390/insects11010049

**Published:** 2020-01-10

**Authors:** Xuping Shentu, Yin Xiao, Yang Song, Zhenyan Cao, Jingxuan Fan, Xiaoping Yu

**Affiliations:** Zhejiang Provincial Key Laboratory of Biometrology and Inspection & Quarantine, College of Life Science, China Jiliang University, Hangzhou 310018, China; stxp@cjlu.edu.cn (X.S.); chiyan17813@sohu.com (Y.X.); songyang@cjlu.edu.cn (Y.S.); S13065707970@163.com (Z.C.); jingxuan_fandry@163.com (J.F.)

**Keywords:** microbial community composition, high-throughput sequencing, comparative bioinformatics, non-fertilized and fertilized eggs, *Nilaparvata lugens* Stål

## Abstract

Yeast-like symbionts (YLSs), harbored in the abdominal fat body of brown planthoppers (BPHs), *Nilaparvata lugens* Stål, play an important role in the growth, development, and reproduction of their host. However, little is known about the diversity of the symbiotic fungal YLSs that are harbored in the eggs of BPHs and the difference between fertilized eggs and non-fertilized eggs. Here, we investigate the fungal community compositions of non-fertilized and fertilized eggs of BPHs and identified the YLSs in the hemolymph by qPCR. A total of seven phyla, 126 genera, and 158 species were obtained from all samples, and Ascomycota and Basidiomycota were the most predominant phyla in the non-fertilized and fertilized eggs. The richness index indicated that microbial diversity in the non-fertilized and fertilized eggs exhibited a profound difference. In addition, 11 strains were only identified in the fertilized eggs, and these strains provide new insights into the constitution of species in YLSs. The difference of *Pichia guilliermondii* in the female hemolymph indicated that fertilization affected the diversity in the eggs by changing the YLSs in the hemolymph. Our research provides a comprehensive understanding of YLS species and their abundance in the eggs of BPHs, and it primarily explores how the changes of YLSs in the hemolymph lead to this difference.

## 1. Introduction

Brown planthopper (BPH), *Nilaparvata lugens* Stål (Hemiptera: Delphacidae), is one of the most destructive monophagous insect pests of rice in Asia [1]. This insect sucks nutrients from the phloem of rice plants and transmits plant viruses, causing the characteristic stunting, wilting, and browning of crops. In recent years, BPH outbreaks have frequently occurred in China and other Asian countries and caused serious rice yield reductions and economic losses [2]. The BPH is a typical monophagous vascular feeder. However, rice phloem sap is an unbalanced diet for BPHs because of its low concentrations of essential amino acids, such as isoleucine, tryptophan, methionine, lysine, histidine, leucine, arginine, and phenylalanine [3,4]. The symbionts that are harbored in the abdominal fat body of BPHs are considered as important resources to maintain nutrition and development.

Yeast-like symbionts (YLSs), which are harbored in the fat body cells of BPH abdomens, are dominant obligatory symbionts [5]. They provide complementary functions, such as essential amino acid synthesis [6], nitrogen storage and recycling [7], steroid synthesis [8], and vitamin supply [9], to their host. Furthermore, a number of YLSs have vital functions in promoting reproduction, and the reduction of YLSs by fungicide propiconazole significantly decreases their emergence rate and number of eggs per female [10]. Previous studies have indicated that the accumulation of microbes in eggs affects oviposition behavior in flies [11], the survival of louse larvae [12], and the presumptive cell fate of *Xiphinema brevicollum* [13]. However, few studies have comprehensively characterized the microbial community in non-embryonated and embryonated eggs in BPHs.

The YLSs in eggs originate from the females of BPHs through transovarial transmission. This process can be elaborated as follows: YLSs in mycetocytes exit the abdominal fat body and are released into the hemolymph by exocytosis. Then, the free YLSs in the hemolymph approach the ovarioles and enter the follicle cells via endocytosis at the epithelial plug of the ovariole. The YLSs congregate at the posterior end of the mature egg after entering and finally form a symbiote ball [14,15]. This transovarial transmission process confirms that the YLSs in eggs originate from the female hemolymph. Therefore, the YLSs in the hemolymph of females could affect the microbial community in their eggs.

Two significant features of YLSs in BPHs can be distinguished from other insects: YLSs in eggs are only obtained from female adults because no YLSs are found in the testes and spermathecae of the mated males, which are totally different from aphids [14,16]. Another feature is that YLSs are also present in non-fertilized eggs, although only the fertilized eggs can hatch into nymphs. Though the YLSs in eggs are only obtained from female BPHs, the release of YLSs from the fat body to the hemolymph could be affected by mating behavior, and fertilization is the key factor in the reproduction of BPHs. However, the effect of fertilization on the transovarial transmission of YLSs has not been studied. The reasons for the differences in the species and amounts of YLS in the eggs of BPHs remain unclear. According to the transovarial transmission process, the YLSs in the hemolymph are important in YLS transport from the abdominal fat body to the eggs. Therefore, evaluating the species that are present in the hemolymph may help to explore why different species in eggs are generated.

In the present work, we analyze the diversity of the microbial communities that are present in the non-fertilized and fertilized eggs of BPHs on the basis of the high-throughput sequencing of the ITS (internal transcribed spacer). This research aimed to determine (i) whether differences exist in the type and abundance of YLSs between the non-fertilized and fertilized eggs of BPHs and, with the use of by qPCR, (ii) the influence of fertilization on YLS species in the hemolymph before entry to eggs. Our research provides a comprehensive understanding of YLS species and their abundance in the eggs of BPHs, and it answers how the changes of YLSs in the hemolymph lead to different species in eggs.

## 2. Materials and Methods 

### 2.1. Insect Mass Rearing and Rice Culture

The BPH population used in the experiments was originally collected from rice fields in Hangzhou (E120°12, N30°16), China. Successive generations were maintained on the susceptible rice variety TN1 in a climatic chamber (26 ± 1 °C, 70%–80% relative humidity, with a 16:8 L:D photoperiod) with soil from the local farm. As soon as the larvae emerged into adults, BPHs were separated by gender to collect their non-fertilized eggs. The TN1 seedlings were cultured in 14 cm-diameter plastic pots and used for BPH mass rearing at the tillering stage (height: 14–16 cm). 

### 2.2. Source of the Fertilized and Non-Fertilized Eggs of BPHs

For the collection of the non-fertilized and fertilized egg masses of the BPHs, groups of synchronized, newly-emerged BPH adults were grouped by 100 females alone and 100 pairs of female and male adults. Then, they were released into separated screen cages, with TN1 rice as the food. The BPHs laid eggs at 72 h after emergence in the leaf sheath and leaf tissue, so the leaves were washed three times in 75% ethanol for 5 min and rinsed twice with sterilized ddH_2_O for three times prior to egg collection. Then, fresh egg masses were collected with sterile needles and forceps for the following experiments. Each treatment was performed in triplicate. The fertilization status of the egg masses was finally confirmed by checking for signs of hatching or embryo development at five days after oviposition. Finally, the collected fertilized and non-fertilized eggs were uniformly placed in 1.5 mL sterile centrifuge tubes. Three samples of non-fertilized egg masses were classified as Group 1 and named UF1, UF2, and UF3 (with UF standing for non-fertilized eggs). Three samples of fertilized egg masses were classified as Group 2 and named F1, F2, and F3 (with F standing for fertilized eggs). All samples were immediately stored under liquid nitrogen until DNA extraction.

### 2.3. Light Microscopy and Comparison of YLS Amount 

The fertilized and unfertilized eggs were collected as above. Their appearance was observed with light microscopy (Nikon SMZ1500, Tokyo, Japan) under 200-fold magnification. The YLSs were released by sticking with a stretched capillary and observed with light microscopy (Nikon Eclipse Ti-S, Tokyo, Japan). Figures were analyzed by using the Nis-Elements software.

The number of YLSs was counted by using a hemocytometer. A group of 330 eggs was polished in 100 μL of PBS buffer (pH 7.4), and the amounts of YLSs were counted and calculated with a hemocytometer. Each experiment was conducted in triplicate.

### 2.4. Total Genomic DNA Extraction 

The total genomic DNA was extracted by using the QIAamp DNA Mini Kit (QIAGEN GmbH, Hilden, Germany) following the manufacturer’s instructions. The quality and quantity of DNA were verified with NanoDrop and 1.0% agarose gels. The extracted DNA was diluted to a concentration of 1 ng/µL and stored at −20 °C until further processing. The diluted DNA was used as a template for polymerase chain reaction (PCR) amplification. 

### 2.5. PCR Amplification

The ITS1 of the fungal ribosomal operon was amplified with universal primers 1743F (5′-CTTGGTCATTTAGAGGAAGTAA-3′) and 2043R (5′-GCTGCGTTCTTCATCGATGC-3′) [17] for fungal diversity analysis. An additional barcode was added to the 1743/2043 primers [18]. The PCR reaction was performed in a 25 µL reaction mixture that contained 1 µL of template DNA, 1 × HiFi PCR buffer, 2.5 mM MgCl_2_, 0.3 mM dNTP for each sample, 0.5 U Taq HiFi HotStart DNA Polymerase (KAPA, Wilmington, MA, USA), and 0.2 µM of each primer. The following PCR cycle conditions were used: Initial denaturation at 95 °C for 3 min followed by 30 cycles at 98 °C for 20 s, 55 °C for 15 s, 72 °C for 45 s, and a final extension at 72 °C for 5 min. 

Amplicon quality was visualized by using 1% gel electrophoresis and purified with Ampure XP beads (Agencourt, Brea, CA, USA) as the template for the second round of reaction. The conditions for the second round of reaction were the same as the first. After purification with Ampure XP beads, the final amplicon was quantified with the Qubit dsDNA Assay Kit (Thermo, Waltham, MA, USA). The samples were pooled at equal concentrations and sequenced through the Illumina MiSeq 250 platform at Shanghai OE Biotech Co., Ltd., Shanghai, China.

### 2.6. Sequence Splicing and Quality Control

The data from Illumina MiSeq sequencing are called raw reads (or raw data). First, ambiguous bases (N) and low-quality sequences were detected and cut off by using the Trimmomatic software (v0.35) [19]. After trimming, paired-end reads were assembled with the FLASH software (v1.2.11) [20]. The parameters of the assembly were as follows: 10 bp minimal overlapping, 200 bp maximum overlapping, and 20% maximum mismatch rate. Sequences were determined for further denoising; reads with ambiguous or homologous sequences or below 200 bp were abandoned. Reads with 75% of bases above Q20 were retained. Then, the reads with chimera were detected and removed. These steps were performed with the QIIME software (v1.8.0) [21]. 

Clean reads were subjected to primer sequence removal and clustering to generate operational taxonomic units (OTUs) by using the UPARSE software with a 97% similarity cutoff [22]. The representative read of each OTU was selected by using the QIIME package. All representative reads were annotated and blasted against the UNITE database by using BLAST. Then, the phylogenetic tree and OTU classification table were finally obtained.

### 2.7. Identification of Pichia Guilliermondii by qPCR 

*Pichia guilliermondii*, which has been proven to be located in the fat body of BPHs by in situ hybridization detection, was selected as the indicator bacteria because of its existence in both the hemolymph and eggs [23]. A group of 30 mated or non-mated female BPHs were collected, respectively. Then, the BPH was frozen with liquid nitrogen, and the chest hemolymph was obtained from the freezing microtome section. The total DNA was extracted from the chest hemolymph as before. The Actin gene was used as the internal reference gene to eliminate the effect of the DNA extraction efficiency difference. The primers used to amplify the Actin gene included Actin-F, GATGAGGCGCAGTCAAAGAG, and Actin-R, GTCATCTTCTCACGGTTGGC. The primers used to identify *P. guilliermondii* were PG-F, TGAAGAACGCAGCGAAAT, and PG-R, AGCAAACGCCTAGTCCG. The quantity of YLSs was the ratio of the YLS copy number and the Actin copy number. The standard curve was calculated by the linear regression of the gene quality and Ct. All the experiments were conducted in triplicate.

### 2.8. Statistical Analysis 

The community ecology metrics, including α-diversity (Chao1, Shannon, Simpson, Good’s coverage, and Specaccum) and β-diversity (principal coordinates analysis (PCoA) and the Bray–Curtis distance method), were calculated by R (v 3.1.2) and QIIME (v 1.7.0). The unweighted pair group method with arithmetic mean (UPGMA) and analysis of similarities (ANOSIM) were also conducted for data analysis. Statistical analysis was performed by using SPSS 20.0, and the differences between groups were analyzed by using the Kruskal–Wallis test.

## 3. Results

### 3.1. Difference of Eggs’ Pattern and YLS Number in Fertilized and Non-Fertilized Eggs

**Figure 1:**
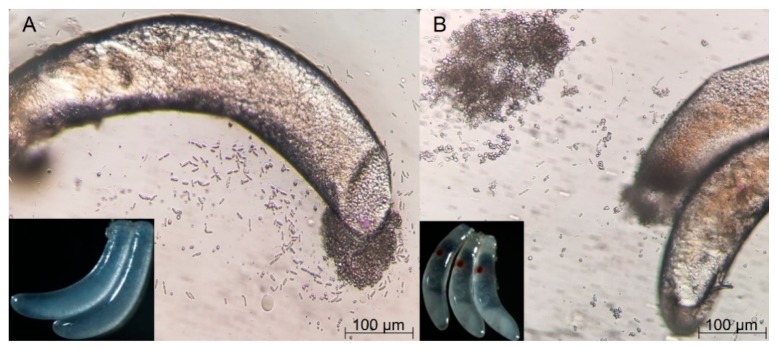
Difference of appearance and Yeast-like symbiont (YLS) number in fertilized and non-fertilized eggs of *Nilaparvata lugens*: (**A**) non-fertilized eggs; (**B**) fertilized eggs.

Though the shape and size of the fertilized and non-fertilized eggs were similar, their appearance and symbionts varied in two situations. On the fertilized eggs, an obvious red point was present at one end, but no such point could be seen on the non-fertilized eggs. Symbionts flowed out after piercing the membrane of eggs under a microscope, proving the existence of YLSs in non-embryonated and embryonated eggs (Figure 1). In addition, the number of YLSs in the fertilized eggs (5417 strains/egg/mL) was much higher than that in the non-fertilized eggs (4583 strains/egg/mL). This phenomenon suggests that fertilization had a profound impact on the vertical transmission. 

### 3.2. Sequencing Results and OTU Analysis

All six samples were successfully amplified by PCR for the ITS1 sequences. After the initial quality control, 216,911 high-quality sequences, including 98,040 sequences from the non-fertilized eggs and 118,871 from the fertilized eggs, were obtained from the six samples. On the basis of a 97% species similarity, 147 and 390 OTUs were obtained from the fertilized and non-fertilized eggs, respectively (Table 1). 

**Figure 2:**
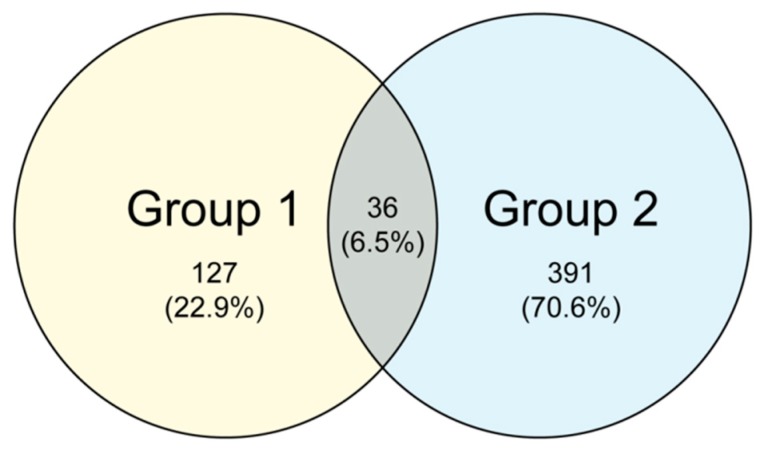
Venn diagram demonstrating the shared and unique operational taxonomic units (OTUs) among the microbiomes of the non-fertilized (Group 1) and fertilized eggs (Group 2) of *N. lugens*.

The common and unique OTUs among the different samples were analyzed with cluster analysis. Figure 2 shows the Venn diagrams of the fungal communities for the non-fertilized and fertilized eggs. A total of 36 common OTUs were observed, accounting for approximately 6.5% of the OTU repertoire, whereas the other 22.9% and 70.6% uniquely existed in the non-fertilized (Group 1) and fertilized eggs (Group 2), respectively. The results show that the fertilized eggs possessed more unique OTUs than the non-fertilized ones.

### 3.3. Composition of the Microbial Community

**Figure 3:**
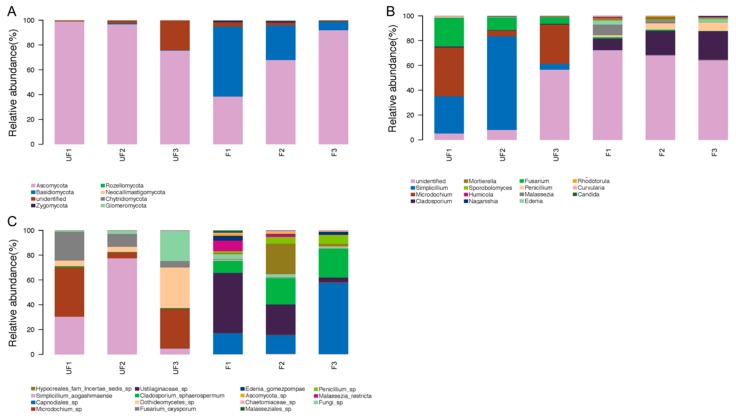
Proportional composition of microbiomes in the non-fertilized and fertilized eggs. (**A**) Composition at the phylum level; (**B**) composition at the genus level; and (**C**) composition at the species level. Groups 1 (including UF1, UF2, and UF3) and 2 (including F1, F2, and F3) represent the non-fertilized and fertilized eggs, respectively.

Seven phyla were detected in all samples, among which four phyla—Ascomycota, Basidiomycota, Rozellomycota, and Zygomycota—were shared in the fertilized and non-fertilized eggs (Figure 3A). Ascomycota and Basidiomycota were the most predominant fungi in the non-fertilized and fertilized eggs, but they differed in abundance. Ascomycota accounted for 88% of the non-fertilized eggs and 66% of the fertilized eggs, whereas Basidiomycota accounted for 30% of the fertilized eggs and 0.5% of the non-fertilized eggs. In addition, Zygomycota and Rozellomycota were detected in both the non-fertilized and fertilized eggs, whereas three unique phyla, namely Chytridiomycota, Glomeromycota, and Neocallimastigomycota, were identified in the non-fertilized eggs. 

At the genus level, 126 genera were detected in all samples, of which the top 15 genera accounted for nearly 97% relative abundance. The genus constitution significantly differed between the non-fertilized and fertilized eggs of *N. lugens*. *Simplicillium*, *Microdochium*, *Fusarium*, and *Cladosporium* were the four most dominant genera in the non-fertilized eggs, accounting for over 70% of the total genera. Conversely, *Cladosporium*, *Penicillium*, *Malassezia*, and *Edenia* were the four most dominant genera in the fertilized eggs, accounting for nearly 30% of the total genus number (Figure 3B).

At the species level, the sequences were classified into 158 species, among which the relative abundances of 15 species accounted for nearly 93% of the total species. *Simplicillium aogashimaense* was the predominant species with the highest percentage, accounting for nearly 40% of the non-fertilized eggs and 90% of the microbial community in the non-fertilized eggs, together with *Microdochium* sp., *Dothideomycetes* sp., and *Fusarium oxysporum*. In the fertilized eggs, the four most dominant genera were *Capnodiales* sp., *Ustilaginaceae* sp., *Cladosporium sphaerospermum*, and *Hypocreales incertae sedis* sp., which accounted for nearly 80% of the total species (Figure 3C).

To obtain evident deviations in microbial richness, microbiomes with relative abundance of over 0.1% were selected for comparative analysis. The shifts in the microbial compositions of the dominant phyla, genera, and species were visualized in the heat map. The composition of the dominant microbiome was assessed by using the Kruskal–Wallis test.

**Figure 4:**
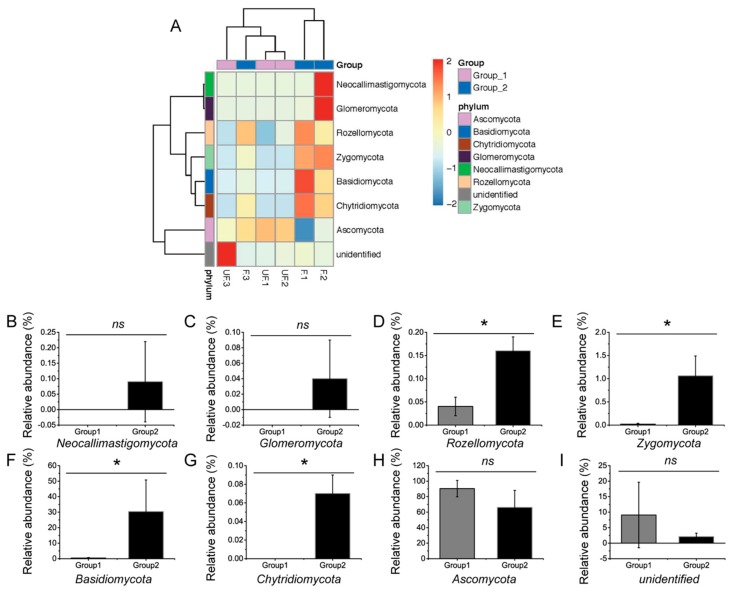
Comparative analysis of the microbial abundance in the dominant phyla. (**A**) Heat map analysis of the dominant phyla in the non-fertilized and fertilized eggs of *N. lugens*. (**B**–**I**) Comparative analysis of the abundance of Basidiomycota, Ascomycota, Chytridiomycota, Glomeromycota, Neocallimastigomycota, Rozellomycota, Zygomycota, and unidentified phylum, respectively. Groups 1 and 2 represent the non-fertilized and fertilized eggs of *N. lugens*, respectively. Ns: no significant difference between the non-fertilized and fertilized eggs at *p* < 0.05 levels; * significant difference between the non-fertilized and fertilized eggs at *p* < 0.05 levels.

At the phylum level, the abundances of Basidiomycota, Chytridiomycota, Rozellomycota, and Zygomycota significantly differed between the fertilized and non-fertilized eggs (*p* < 0.05) (Figure 4A–I), whereas those of Ascomycota, Glomeromycota, and Neocallimastigomycota demonstrated no significant difference (*p* = 0.127, 0.317, and 0.317, respectively).

**Figure 5:**
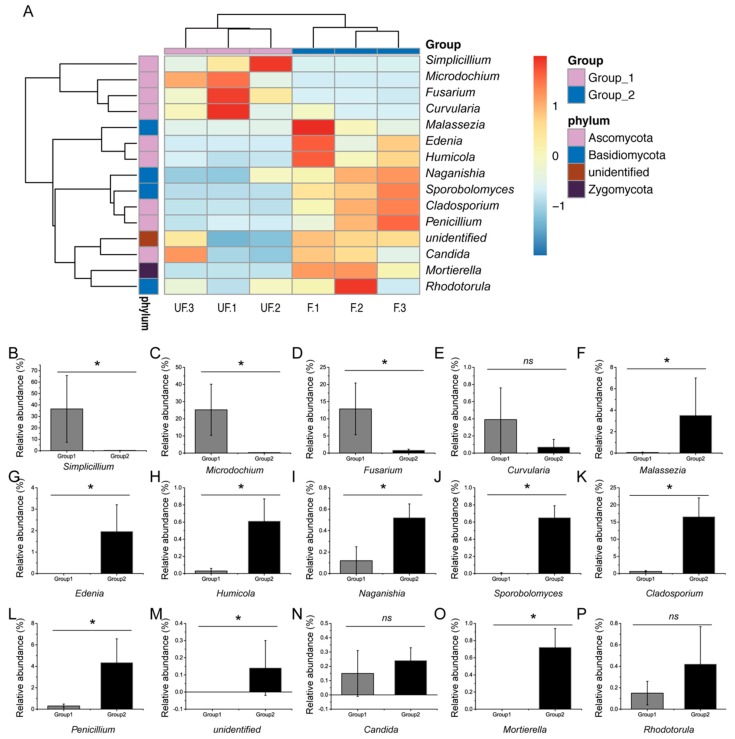
Comparative analysis of microbial abundance in dominant genera. (**A**) Heat map analysis of the dominant phyla in the non-fertilized and fertilized eggs of *N. lugens*. (**B**–**P**) Comparative analysis of abundance in the genera of *Simplicillium*, *Microdochium*, *Fusarium*, *Curvularia*, *Malassezia*, *Edenia*, *Humicola*, *Naganishia*, *Sporobolomyces*, *Cladosporium*, *Penicillium*, unidentified genus, *Candida*, *Mortierella*, and *Rhodotorula*, respectively. Groups 1 and 2 represent the non-fertilized and fertilized eggs of *N. lugens*, respectively. Ns: no significant difference between the non-fertilized and fertilized eggs at *p* < 0.05 levels; * significant difference between the non-fertilized and fertilized eggs at *p* < 0.05 levels.

At the genus level, 80% of the dominant genera, including *Simplicillium*, *Microdochium*, *Fusarium*, *Malassezia*, *Phoma*, *Edenia*, *Humicola*, *Sporobolomyces*, *Acremonium*, *Aspergillus*, *Penicillium*, *Cladosporium*, *Naganishia*, *Ceratobasidium*, and *Mortierella*, exhibited significant differences between the relative abundance of the fertilized and non-fertilized eggs. The relative abundance of three genera, including *Curvularia*, *Candida*, and *Rhodotorula* (*p* = 0.13, 0.51, and 0.28, respectively), demonstrated no significant difference (Figure 5A–P).

### 3.4. Comparisons of Microbial Community Structure 

Table 2 shows the calculated Chao1, Shannon, and Good’s coverage for the non-fertilized and fertilized eggs. The diversity and richness of the total microbial communities were measured by using Shannon and Chao1 values, respectively (Table 2). The sample completeness was analyzed with the use of Good’s coverage. The microbial richness index (Chao1) of the fertilized eggs was higher than that of the non-fertilized eggs (*p* < 0.05), indicating that the number of species in the microbial community of the fertilized eggs was higher than that in the non-fertilized eggs. However, the microbial diversity index values (Shannon) of the fertilized and non-fertilized eggs were similar (*p* = 0.07), indicating that no significant difference existed between the species and uniformity of fungal distribution among the microbial communities of the fertilized and non-fertilized eggs. Meanwhile, the results of Good’s coverage, which is an estimator of sampling completeness, indicated a good overall sampling with levels of >99.8% (Table 2). A rarefaction curve analysis showed that all samples were almost parallel to the x-axis, indicating that the obtained reads were sufficient to represent the overall fungal diversity (Figure S1).

### 3.5. Comparative Bioinformatics Analyses between the Non-Fertilized and Fertilized Eggs

**Figure 6:**
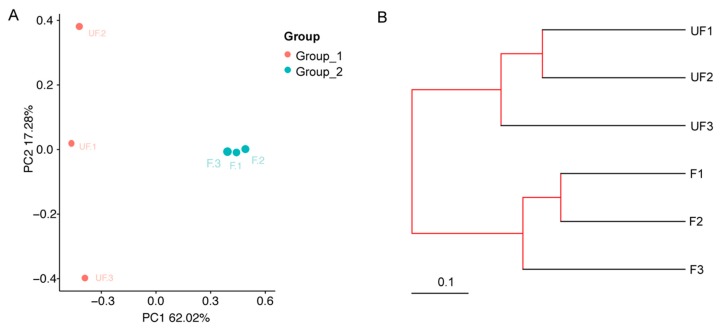
β-diversity analysis for the non-fertilized and fertilized eggs. (**A**) Principal coordinates analysis (PCoA) plots on the basis of weighted UniFrac distance matrices. (**B**) Unweighted pair group method with arithmetic mean (UPGMA) clustering on the basis of the unweighted UniFrac distance analysis.

The microbial community structure relationships between the non-fertilized and fertilized eggs were visualized by using phylogenetic beta indexes. On the basis of the PCoA plot (the Bray–Curtis distance method), the non-fertilized egg microbiome (Group 1) was separately clustered from the fertilized eggs (Group 2) through the first principal coordinate (Figure 6A). In addition, the F1, F2, and F3 close proximities showed similar microbial species, whereas UF1, UF2, and UF3 were dispersed, indicating that the composition of the microbial community structures of these samples differed.

By using UPGMA clustering on the basis of unweighted UniFrac distance analysis, UF1–3 and F1–3 formed clusters I and II, respectively (Figure 6B). Evidently, the samples from different groups [Group 1 (UF1–3) or 2 (F1–3)] exhibited cross clustering in clusters I and II, indicating that the microbial community structures of the non-fertilized and fertilized eggs were significantly different. In the bacterial ANOSIM between the non-fertilized (Group 1) and fertilized eggs (Group 2), the R and *p* values were 1 (close to 1) and 0.097, respectively, indicating that the intergroup differences were greater than the intra-group differences for the non-fertilized and fertilized eggs (Table S1).

### 3.6. Different Species of YLS in the Fertilized and Non-Fertilized Eggs

In the analysis of the diversity of the microbial community, 28 OTUs were identified by other researchers, and five species, including *S. aogashimaense*, *Hypocreales* fam *Incertae sedis* sp., *F. oxysporum*, *Fusarium* sp., and *Candida parapsilosis*, were the dominant fungi in the YLSs. However, by comparison of the strains in the fertilized and non-fertilized eggs, 11 strains, including *Candida metapsilosis*, *P. guilliermondii*, *Saccharomycetales* sp., *Saccharomycetes*, *Kazachstania pintolopesii*, *Acremonium implicatum*, *Trichoderma longibrachiatum*, *Fusarium* sp., *Nectriaceae* sp., *Hypocreales* sp., and *Sarocladium strictum*, were identified in the fertilized eggs.

### 3.7. Verification of Different Species in the Hemolymph

*P. guilliermondii*, which has been proven to be located in the fat body of BPHs by in situ hybridization detection, was selected as the indicator bacteria to prove, via qPCR, the release of YLSs from the fat body to the hemolymph because of its existence in both the hemolymph and eggs [23]. *P. guilliermondii* could be detected 36 h after emergence but not in the hemolymph in non-mated females (Table 3). This result indicated that fertilization changed the constitution of YLSs in the hemolymph, which may have led to the difference of diversity in the fertilized and non-fertilized eggs.

## 4. Discussion

YLSs are the most important symbiotic strains in *N. lugens*; they could compensate for unbalanced amino acids, the recycling of uric acid, and vitamin biosynthesis pathway for its host. This study proves, for the first time, the existence of YLSs in non-fertilized eggs and has determined the effect of fertilization on vertical transmission. In addition, the YLSs were identified by an advanced ITS sequencing method. At present, numerous methods have been used to identify YLS species and compositions. *Hypomyces chrysospermus* was isolated and identified from the abdominal fat of *N. lugens* by centrifugation and 18S rDNA sequencing [24]. Two cultured strains belonging to *Yarrowia lipolytica* and *Sterigmatomyces halophilus* were identified by using the 26S rDNA sequences [25]. *P. guilliermondii*, *Cryptococcus* sp., and *Candida quercitrusa* were identified with sequencing analysis of the 18S rDNA and ITS-5.8S rDNA [23,26]. *Saccharomycetales* sp., *Debaryomyces hansenii*, and several uncultured fungi were detected by using a novel method of nested PCR–denaturing gradient gel electrophoresis (DGGE) [27]. Though the sequencing of 18S rDNA and 26S rDNA and DGGE were applied in the taxonomic status identification of fungi, previous studies on *N. lugens* have shown comparison results on the differences in the complete sequences of 18S rDNA and ITS-5.8S rDNA of YLS from *N. lugens*, suggesting that different sequencing methods yield different analysis results on species relationships [28]. 18S rDNA is a highly conserved sequence, whereas the ITS is a moderately conserved sequence that exhibits considerable inter- and intraspecific differences and provides rich information for the taxonomic classification and molecular detection of fungi [29]. Thus, high-throughput sequencing based on the ITS could obtain more detailed YLS information.

The present study is the first to provide insights into the detailed composition and comparison of YLS in *N. lugens* eggs via the high-throughput sequencing of the ITS. We identified 11 strains in the fertilized eggs only. YLSs play important roles in the growth and reproduction of *N. lugens*. Previous studies have shown that the inhibition of YLSs in the fat body of *N. lugens* by pesticides could lead to a decreased reproduction rate. In addition, the constitution of YLSs in *N. lugens* could result in different resistances to rice [27]. However, the comprehensive diversity of YLS in *N. lugens* eggs has not been detected and compared until now. In this study, through the comparative analysis of the diversity of microbial communities between the non-fertilized and fertilized eggs of *N. lugens*, significant differences were observed in the species abundance between the non-fertilized and fertilized eggs. However, the composition of most species in each taxonomic category showed no significant difference. Eleven strains were identified in the fertilized eggs only. To explain the reasons for the difference of diversity in fertilized and non-fertilized eggs, we analyzed *P. guilliermondii* with quantitative PCR of the hemolymph of the BPH and noted that the organism could be detected only in mated female BPHs, indicating that it is not released from the fat body of non-mated female BPHs by exocytosis. However, the species of YLSs and their corresponding functions remain unclear because of the uncultured characteristics of YLSs. These strains may be used to study the intimate relationship between YLSs and BPHs and to clarify their functions in BPH reproduction. 

Many reasons could lead to the different releases of YLS from the fat body. The juvenile hormone (JH), a sesquiterpenoid hormone that is synthesized and secreted by the corpus allatum, is one of the most important hormones that is changed by fertilization [30]. The JH titer in female adults significantly increases after mating because of the transfer from male adults to female adults during mating. JH regulates various biological activities in insects, including development, metamorphosis, and reproduction. The molecular mechanisms of JH action in insect reproduction, including vitellogenesis, oocyte maturation, and ovarian growth, remain largely unknown [1]. However, we speculate that fertilization possibly causes the an increase in the JH titer in BPH female adults, resulting in the exocytosis of YLSs from the fat body into the hemolymph. Therefore, JH may also regulate the differences in YLS species and their abundance in fertilized and non-fertilized eggs. Related experiments are required in the future to verify the JH-regulated release of YLSs from the fat body of BPHs. We believe that YLSs are the key to understanding the mechanism of JH action in BPH reproduction because of their importance to hosts.

In conclusion, the microbial community structures in fertilized and non-fertilized eggs provide important information on transovarial transmission. These results indicate that fertilization changes the diversity of the microbial community in eggs. Eleven strains were detected in the fertilized eggs only, and *Pichia guilliermondii* was only detected in the female hemolymph after fertilization, suggesting that fertilization changes diversity in eggs by altering the hemolymph. Though a distinct relationship exists between fertilization and hormones, further research on the mechanism of hormones on the YLS transport and functional profile of microbiome will provide information on the transovarial transmission of YLSs in *N. lugens*.

## Figures and Tables

**Table 1:** Sequencing and analysis results of the non-fertilized and fertilized eggs.

Sample ID	Valid Tags	Mean Length (bp)	OUT Counts
UF.1	15,339	252.22	26
UF.2	41,375	268.55	59
UF.3	41,326	252.65	62
Total	98,040		147
F.1	39,495	288.96	123
F.2	39,194	272.07	168
F.3	40,182	254.65	99
Total	118,871		390

Note: UF: non-fertilized eggs; F: fertilized eggs.

**Table 2:** α-diversity analysis of non-fertilized and fertilized eggs.

Sample ID	Chao1	Shannon	Good’s Coverage
UF.1	38	2.0160	0.9994
UF.2	65	1.5003	0.9991
UF.3	67	2.5542	0.9992
F.1	134	3.3055	0.9992
F.2	177	3.7759	0.9987
F.3	113	2.4978	0.9991

**Table 3:** Identification of *Pichia guilliermondii* in the chest hemolymph of non-mated and mated BPH females.

Condition	Time after Emergence						
	24 h	32 h	40 h	48 h	56 h	64 h	72 h
Non-mated female BPH	0.000	0.000	0.000	0.000	0.000	0.000	0.000
Mated female BPH	0.000 ^a^	0.357 ± 0.184 ^a^	0.321 ± 0.107 ^a^	0.306 ± 0.093 ^a^	1.431 ± 0.819 ^a^	1.408 ± 0.708 ^a^	7.955 ± 1.623 ^b^

‘^a,b^’ in the same line indicate significant differences at 5% level.

## Supplementary Materials

The following are available online at https://www.mdpi.com/2075-4450/11/1/49/s1, Figure S1: Rarefaction curve analysis of fertilized eggs and non-fertilized eggs of Good’s coverage, Table S1: The Anosim analysis by calculating the distance of Binary Curtis.

## References

[1] Sogawa K., Cheng C.H., Pathak M.D. (1979). Economic thresholds, nature of damage, and losses caused by the brown planthopper. Brown Planthopper: Threat to Rice Production in Asia, Chapter 7.

[2] Wu S.F., Zeng B., Zheng C., Mu X.C., Zhang Y., Hu J., Zhang S., Gao C.F., Shen J.L. (2018). The evolution of insecticide resistance in the brown planthopper (*Nilaparvata lugens* Stål) of China in the period 2012–2016. Sci. Rep..

[3] Fukumorita T., Chino M. (1982). Sugar, amino acid and inorganic contents in rice phloem sap. Plant Cell Physiol..

[4] Ohshima T., Hayashi H., Chino M. (1990). Collection and chemical composition of pure phloem sap from *Zea mays* L.. Plant Cell Physiol..

[5] Tang M., Lv L., Jing S., Zhu L., He G. (2010). Bacterial symbionts of the brown planthopper, *Nilaparvata lugens* (Homoptera: Delphacidae). Appl. Environ. Microb..

[6] Fan H.W., Noda H., Xie H.Q., Suetsugu Y., Zhu Q.H., Zhang C.X. (2015). Genomic analysis of an Ascomycete fungus from the rice planthopper reveals how it adapts to an endosymbiotic lifestyle. Genome Biol. Evol..

[7] Hongoh Y., Sasaki T., Ishikawa H. (2016). Cloning, sequence analysis and expression in Escherichia coli of the gene encoding a uricase from the yeast-like symbiont of the brown planthopper, *Nilaparvata lugens*. Insect Biochem. Mol. Biol..

[8] Noda H., Koizumi Y. (2003). Sterol biosynthesis by symbiotes: Cytochrome P450 sterol C-22 desaturase genes from yeast-like symbiotes of rice planthoppers and anobiid beetles. Insect Biochem. Mol. Biol.

[9] Xue J., Zhou X., Zhang C.X., Yu L., Fan H., Wang Z., Xu H.J., Xi Y., Zhu Z., Zhou W. (2014). Genomes of the rice pest brown planthopper and its endosymbionts reveal complex complementary contributions for host adaptation. Genome Biol..

[10] Shentu X.P., Wang X.L., Xiao Y., Yu X.P. (2019). Effects of fungicide propiconazole on the yeast-like symbiotes in brown planthopper (BPH, *Nilaparvata lugens* Stål), and its role in controlling BPH Infestation. Front. Physiol..

[11] Lam K., Babor D., Duthie B., Babor E.M., Moore M., Gries G. (2007). Proliferating bacterial symbionts on house fly eggs affect oviposition behaviour of adult flies. Anim. Behav..

[12] Aschner M. (1934). Studies on the symbiosis of the body louse: I. Elimination of the symbionts by centrifugalisation of the eggs. Parasitology.

[13] Coomans A., Claeys M., Vandekerckhove T.T. (2000). Transovarial transmission of symbionts in *Xiphinema brevicollum* (Nematoda: Longidoridae). Nematology.

[14] Cheng D.J., Hou R.F. (2001). Histological observations on transovarial transmission of a yeast-like symbiote in *Nilaparvata lugens* Stål (Homoptera, Delphacidae). Tissue Cell.

[15] Yukuhiro F., Miyoshi T., Noda H. (2014). Actin-mediated transovarial transmission of a yeast-like symbiont in the brown planthopper. J. Insect Physiol..

[16] Liljesthröm G., Brentassi M.E. (2016). Modeling population dynamics of yeast-like symbionts (Ascomycota, Pyrenomycetes, Clavicipitaceae) of the planthopper *Delphacodes kuscheli* (Hemiptera: Delphacidae). Symbiosis.

[17] Bellemain E., Carlsen T., Brochmann C., Coissac E., Taberlet P., Kauserud H. (2010). ITS as an environmental DNA barcode for fungi: An in silico approach reveals potential PCR biases. BMC Microbiol..

[18] Hamady M., Walker J.J., Harris J.K., Gold N.J., Knight R.J. (2008). Error-correcting barcoded primers for pyrosequencing hundreds of samples in multiplex. Nat. Methods.

[19] Bolger A.M., Marc L., Bjoern U. (2014). Trimmomatic: A flexible trimmer for Illumina sequence data. Bioinformatics.

[20] Reyon D., Tsai S.Q., Khayter C., Foden J.A., Sander J.D., Joung J.K. (2012). FLASH assembly of TALENs for high-throughput genome editing. Nat. Biotechnol..

[21] Caporaso J.G., Kuczynski J., Stombaugh J., Bittinger K., Bushman F.D., Costello E.K., Fierer N., Peña A.G., Goodrich J.K., Gordon J.I. (2010). QIIME allows analysis of high-throughput community sequencing data. Nat. Methods.

[22] Edgar R.C., Haas B.J., Clemente J.C., Christopher Q., Rob K. (2011). UCHIME improves sensitivity and speed of chimera detection. Bioinformatics.

[23] Dong S.Z., Pang K., Bai X., Yu X.P., Hao P.Y. (2011). Identification of two species of yeast-like symbiotes in the Brown Planthopper, *Nilaparvata lugens*. Curr. Microbiol..

[24] Noda H., Nakashima N., Koizumi M. (1995). Phylogenetic position of yeast-like symbiotes of rice planthoppers based on partial 18S rDNA sequences. Insect Biochem. Mol. Biol..

[25] Zhang J.F., Wu H., Chen J.M., Zheng X.S., Chen L.Z., Yu X.P. (2007). A strain isolated from brown planthopper and its molecular identification. Chin. J. Rice Sci..

[26] Pang K., Dong S.Z., Hou Y., Bian Y.L., Yang K., Yu X.P. (2012). Cultivation, identification and quantification of one species of yeast-like symbiotes, *Candida*, in the rice brown planthopper, *Nilaparvata lugens*. Insect Sci..

[27] Hou Y., Ma Z., Dong S.Z., Chen Y.H., Yu X.P. (2013). Analysis of yeast-like symbiote diversity in the brown planthopper (BPH), *Nilaparvata lugens* Stål, using a novel nested PCR-DGGE protocol. Curr. Microbiol..

[28] Zhou Y.Y., Dong S.Z., Bai X., Yu X.P. (2012). Phylogenetic position of yeast-like symbiotes from three rice planthoppers (Homoptera:Delphacidae) based on 18S rDNA and ITS-5.8S rDNA sequences. Acta Entomol. Sin..

[29] Anderson I.C., Campbell C.D., Prosser J.I. (2010). Potential bias of fungal 18S rDNA and internal transcribed spacer polymerase chain reaction primers for estimating fungal biodiversity in soil. Environ. Microbiol..

[30] Bright M., Bulgheresi S. (2010). A complex journey: Transmission of microbial symbionts. Nat. Rev. Microbiol..

