# Supplementary material for: Comparative Analysis of the Diversity of the Microbial Communities between Non-Fertilized and Fertilized Eggs of Brown Planthopper, Nilaparvata lugens Stål
Source: Insects. 2020 Jan 10;11(1):49. doi: 10.3390/insects11010049 (PMC7022379; doi:10.3390/insects11010049)
Supplement: Supplementary file 1 [file insects-11-00049-s001.pdf]

Comparative analysis of the diversity of the microbial communities between non-fertilized and fertilized eggs of brown planthopper, *Nilaparvata lugens* Stål

Xuping Shentu[1], Yin Xiao[1], Yang Song, Zhenyan Cao, Jingxuan Fan and Xiaoping Yu*

Zhejiang Provincial Key Laboratory of Biometrology and Inspection & Quarantine, College of Life Science, China Jiliang University, Hangzhou 310018, China

[1] Contributed equally to this article

* Correspondence: yxp@cjlu.edu.cn; Tel.: +86-571-86836006; Fax: +86-571-86836006

Figure S1 Rarefaction curve analysis of fertilized eggs and non-fertilized eggs of Good's coverage. The non-fertilized eggs included UF1, UF2 and UF3. The fertilized eggs included F1, F2 and F3.

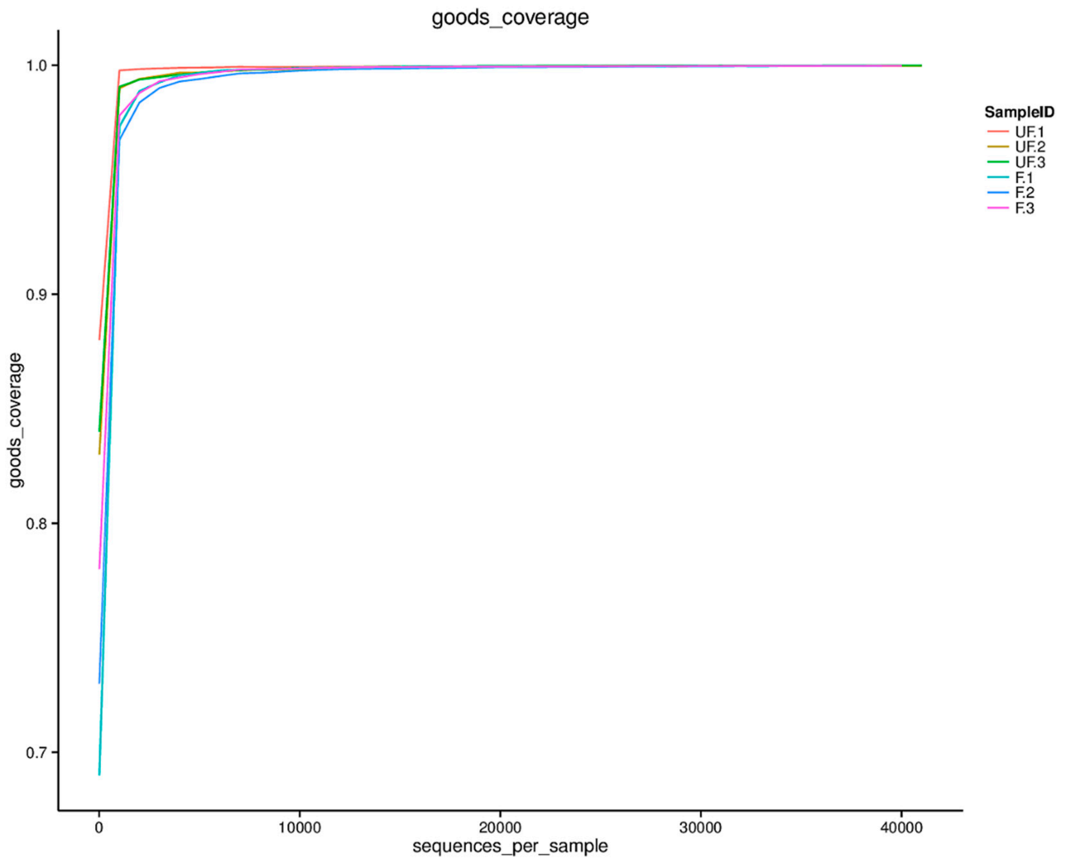

Table S1 The Anosim analysis by calculating the distance of Binary Curtis

Method	R statistic	*p*-value	Number of permutations
Anosim	1.0000	0.097	999

R: the difference between groups. The R near 1 indicated the difference between groups was more obvious than the difference in groups.
